# Supplementary material for: Switching from Electron to Hole Transport in Solution-Processed Organic Blend Field-Effect Transistors
Source: Polymers (Basel). 2020 Nov 11;12(11):2662. doi: 10.3390/polym12112662 (PMC7709025; doi:10.3390/polym12112662)
Supplement: Supplementary file 1 [file polymers-12-02662-s001.zip › polymers-927762-supplementary.docx]

**Supporting information**

**Switching from electron to hole transport in solution-processed organic blend field-effect transistors**

Julia Fidyk ^1^, Witold Waliszewski ^1^, Piotr Sleczkowski ^1^, Adam Kiersnowski ^2,3^,
Wojciech Pisula ^1,4^ and Tomasz Marszalek ^1,4,^*

^1^ Department of Molecular Physics, Faculty of Chemistry, Lodz University of Technology, Zeromskiego 116, 90-924 Lodz, Poland; julia.fidyk@dokt.p.lodz.pl (J.F.); witold.waliszewski@dokt.p.lodz.pl (W.W.); piotr.sleczkowski@p.lodz.pl (P.S.); pisula@mpip-mainz.mpg.de (W.P.)

^2^ Leibniz Institute of Polymer Research, Hohe Str. 6, 01069 Dresden, Germany; kiersnowski@ipfdd.de

^3^ Wroclaw University of Science and Technology, Wybrzeze Wyspianskiego 27, 50-370 Wroclaw, Poland

^4^ Max Planck Institute for Polymer Research, Ackermannweg 10, 55128 Mainz, Germany

**
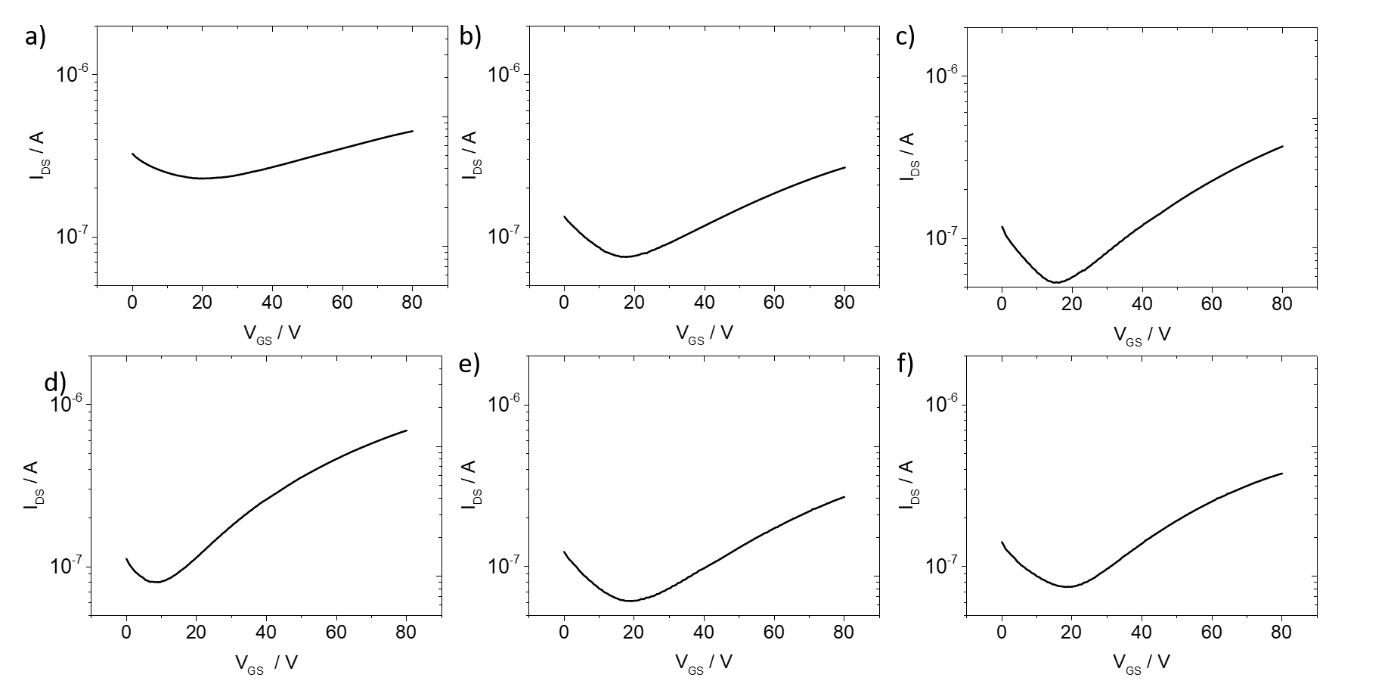
**

Figure S1. Transfer characteristics (V_DS_ = +80V) of OFETs based on PBTTT-C_14_:PDI8-CN_2_ composites obtained by spin-coating for 100 °C solution and: a) 1000 rpm, b) 2000 rpm, c) 3000 rpm, d) 4000 rpm, e) 5000 rpm, f) 6000 rpm rotation speeds

Table S1. Field-effect mobility values for PBTTT-C_14_:PDI8-CN_2_ composites obtained by spin-coating for 100 °C solution and different rotation speeds.

| Rotation speed [RPM] | 1000 | 2000 | 3000 | 4000 | 5000 | 6000 |
| --- | --- | --- | --- | --- | --- | --- |
| Thickness [nm] | 80 | 68 | 42 | 55 | 53 | 38 |
| Channel length [μm] | 25 | 30 | 30 | 30 | 30 | 25 |
| Field-effect mobility  [10^-4^ cm^2^/(Vs)] | 0.8 | 1.0 | 2.1 | 2.3 | 1.3 | 1 |

**
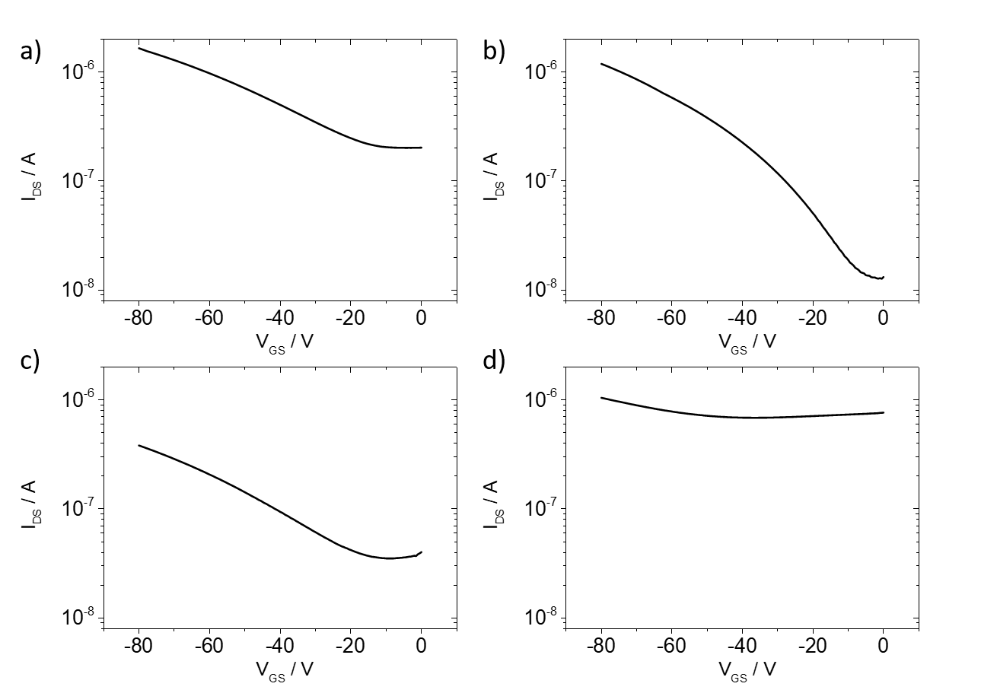
**

Figure S2. Transfer characteristic (V_DS_ = -80V) of OFETs based on PBTTT-C_14_:PDI8-CN_2_ composites fabricated with rotation speed of 4000 rpm and solutions at a) 80 °C , a) 100 °C , a) 120 °C , a) 140 °C.

Table S2. Field-effect mobility values for PBTTT-C_14_:PDI8-CN_2_ composites fabricated with rotation speed of 4000 rpm and different solution temperatures.

| Solution temperature [°C] | 80 | 100 | 120 | 140 | 160 | 180 |
| --- | --- | --- | --- | --- | --- | --- |
| Field-effect mobility [10^-3^cm^2^/(Vs)] | 0.5 | 1.0 | 0.3 | 0.2 | - | - |


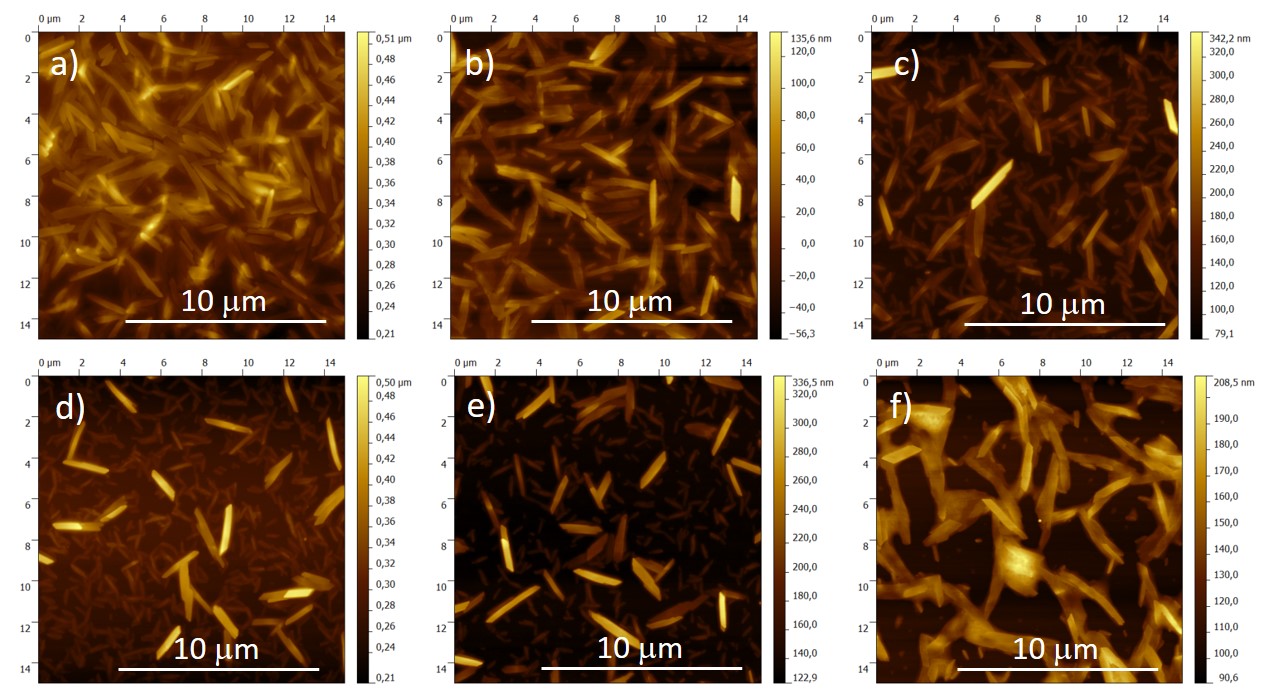


Figure S3. AFM images of solution temperature dependence for PBTTT-C_14_:PDI8-CN_2_ heterojunction composites additionally annealed at 200°C. PBTTT-C_14_:PDI8-CN_2_ films obtained at: a) 80 °C, b) 100 °C, c) 120 °C, d) 140 °C, e) 160 °C, f) 180 °C.


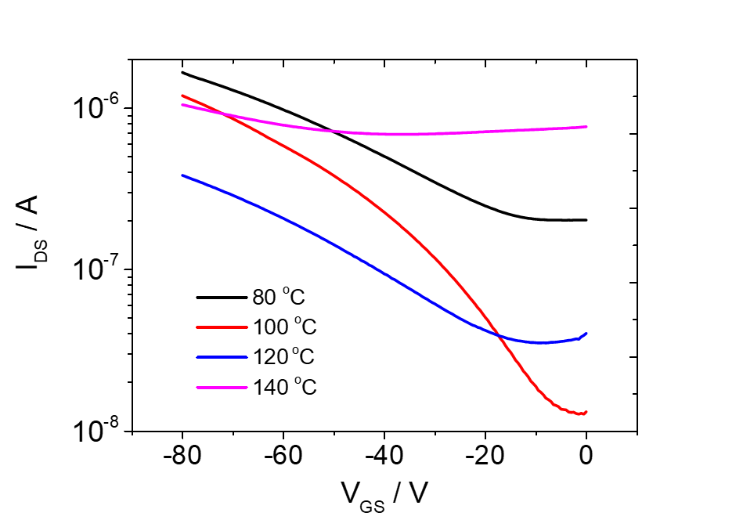


Figure S4. Transfer characteristic (V_DS_ = -80V) of OFETs based on PBTTT-C_14_:PDI8-CN_2_ composite with rotation speed of 3000 for various solution temperature after annealing at 140 °C


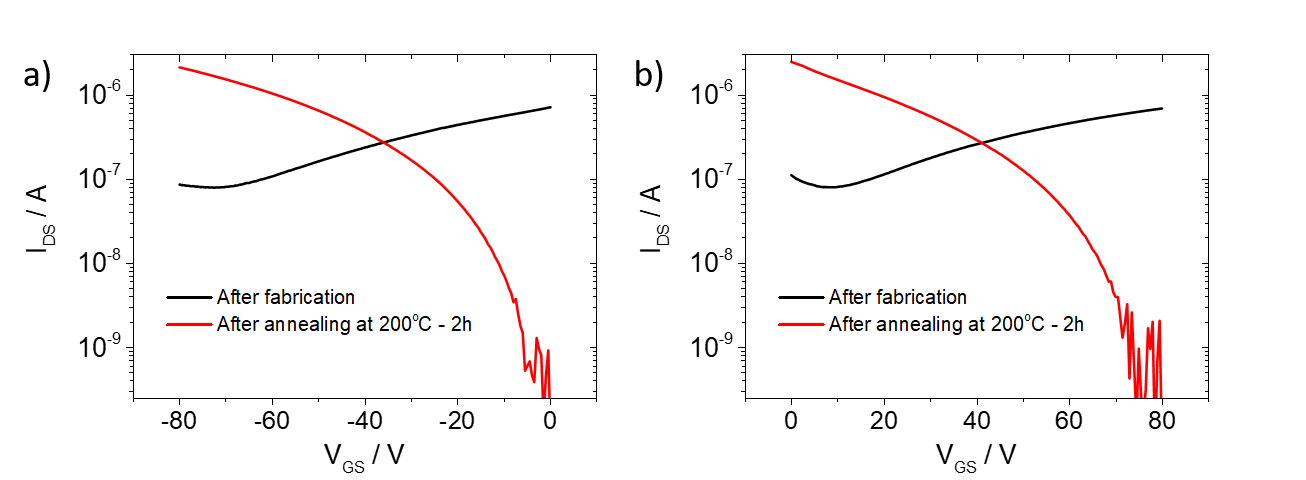


Figure S5. Transfer characteristic of OFETs based on PBTTT-C_14_:PDI8-CN_2_ composite with rotation speed of 4000 rpm a) *p-type* (V_DS_ = -80V) and b) *n-type* (V_DS_ = +80V) behavior.


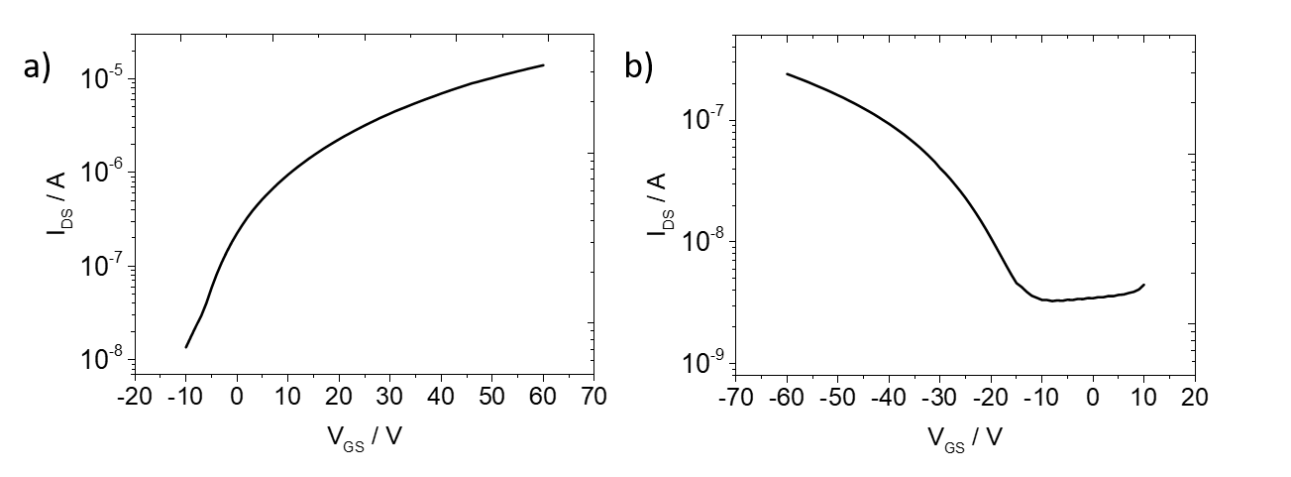


Figure S6. Transfer characteristic of OFETs based on PBTTT-C_14_:PDI8-CN_2_ composite fabricated with rotation speed of 4000 rpm on substrate modified by OTS; a) before annealing - *n*-type *type* (V_DS_ = +60V) b) annealed at 200 °C - *p-type* (V_DS_ = -60V)

Table S3. Field-effect mobility values for PBTTT-C_14_:PDI8-CN_2_ composites fabricated with rotation speed of 4000 rpm on substrate modified by OTS.

| Thermal annealing | None | | 200°C/2h | |
| --- | --- | --- | --- | --- |
| Transport type | *p-type* | *n-type* | *p-type* | *n-type* |
| Field-effect mobility [10^-2^ cm^2^/(Vs)] | - | 1.5 | 0.1 | - |

|    |  |    |
| --- | --- | --- |
|  | (d) |  |
|    |  |    |

Figure S6. Vertical (q­_z_ / vert. – upper plots) and horizontal (q_x,y_, horiz. – lower plots) integrations of GIWAXS patterns shown in Figure 6 in the main part of the paper. The integrations correspond to PBTTT-C_14_ (a), annealed PBTTT-C_14_ (b), PBTTT-C_14_:PDI8-CN_2_ blend (c), and the annealed PBTTT-C_14_:PDI8-CN_2_ (d). In the horizontal integrations of PBTTT-C_14_ the deconvolution of patterns is included. Open points correspond to the experimental data, color lines show contributions of the π-π-stacking components and amorphous halos whereas black solid line correspond to the fitted data. If not specified otherwise Miller hkl indices correspond to crystal structure of PBTTT-C_14_.

(c)

(a)

(b)

Table S4. PBTTT-C_14_ crystal structure parameters extracted from GIWAXS data shown in Figure SX. q_max_, d, and t denote, respectively, peak position, interplanar spacing and crystal coherence length (Scherrer coherence) 100 and π-π subscripts correspond to 100 interplanar distance and π-π stacking distance. Since in PBTTT-C_14_ there are two distinct π-π systems (see main text for details) there are
π-π_1_ and π-π_2_ symbols.

| Crystal structure parameter | **System** | | | |
| --- | --- | --- | --- | --- |
|  | PBTTT-C_14_ | PBTTT-C_14­_,  annealed | PBTTT-C_14_:PDI8-CN_2_ | PBTTT-C_14_:PDI8-CN_2_  annealed |
| q_­max (100)_ / Å^-1^ | 0.276 | 0.276 | 0.284 | 0.274 |
| d_100 ­_/ Å | 22.8 | 22.8 | 22.2 | 22.9 |
| t_100_ / Å | 115 | 170 | 90.9 | 180 |
| q_­max (_π-π_1) /_ Å^-1^ | 1.44 | 1.44 | n/a | n/a |
| d_­π-π1_ / Å | 4.4 | 4.4 | n/a | n/a |
| d_­π-π1_/ Å | 19.4 | 26.8 | n/a | n/a |
| q_­max (_π-π_2) /_ Å^-1^ | 1.64 | 1.64 | n/a | n/a |
| d_π-π2_ / Å | 3.8 | 3.8 | n/a | n/a |
| t_­π-π2_ / Å | 22.5 | 22.5 | n/a | n/a |
